# Errata

**Published:** 2013-03-01

## Vol. 61, Nos. 51 & 52

On page ND-719, in “Table I. Provisional cases of infrequently reported notifiable diseases (<1,000 cases reported during the preceding year) — United States, week ending December 29, 2012 (52nd week),” in the row “Diphtheria,” under the column heading “Cum 2012,” the value should read, “**1**.”

On page ND-730, in “Table II. (*Continued*) Provisional cases of selected notifiable diseases, United States, weeks ending December 29, 2012, and December 31, 2011 (52nd week),” multiple errors occurred under the heading, “*Streptococcus pneumoniae*, invasive disease.” The corrected table section, with new values shown in **bold**, is as follows.

**TABLE II:** (*Continued*) Provisional cases of selected notifiable diseases, United States, weeks ending December 29, 2012, and December 31, 2011 (52nd week)*

Reporting area	*Streptococcus pneumoniae*,† invasive disease									

	All ages					Age <5				
	
	Current week	Previous 52 weeks		Cum 2012	Cum 2011	Current week	Previous 52 weeks		Cum 2012	Cum 2011
	
		Med	Max				Med	Max		
**United States**	106	241	803	**13,160**	**17,138**	7	20	58	**1,002**	**1,273**
**New England**	3	11	24	579	807	—	1	4	50	54
Connecticut	1	5	13	280	354	—	0	2	14	14
Maine	—	2	7	94	136	—	0	1	3	4
Massachusetts	—	1	3	42	38	—	0	2	26	19
New Hampshire	—	1	5	62	110	—	0	1	6	5
Rhode Island	—	0	5	42	97	—	0	1	1	5
Vermont	2	1	4	59	72	—	0	0	—	7
**Mid. Atlantic**	17	36	157	1,902	2,598	—	2	11	102	138
New Jersey	—	7	26	408	680	—	0	3	23	43
New York (Upstate)	11	17	108	856	1,183	—	1	10	55	56
New York City	6	13	24	638	735	—	0	2	24	39
Pennsylvania	N	0	0	N	N	N	0	0	N	N
**E.N. Central**	27	54	101	2,694	3,283	1	4	10	174	186
Illinois	N	0	0	N	N	—	0	0	—	—
Indiana	—	12	33	570	819	—	1	2	33	40
Michigan	2	12	25	529	694	—	0	3	31	36
Ohio	20	21	46	1,150	1,278	—	2	6	86	83
Wisconsin	5	8	29	445	492	1	0	2	24	27
**W.N. Central**	—	14	58	679	**835**	—	1	4	53	**77**
Iowa	N	0	0	N	N	N	0	0	N	N
Kansas	N	0	0	N	N	N	0	0	N	N
Minnesota	—	9	22	455	580	—	0	3	31	47
Missouri	N	0	0	N	**N**	—	0	0	—	—
Nebraska	—	2	8	106	121	—	0	2	10	12
North Dakota	—	0	38	30	91	—	0	2	1	4
South Dakota	—	1	5	88	**43**	—	0	2	11	**14**
**S. Atlantic**	32	56	193	**2,968**	4,009	4	4	21	232	343
Delaware	—	0	3	31	52	—	0	1	1	—
District of Columbia	—	0	4	45	55	—	0	1	3	6
Florida	30	18	48	989	1,324	4	1	8	80	138
Georgia	—	15	42	866	1,173	—	2	4	76	94
Maryland	2	6	22	**331**	587	—	0	5	27	51
North Carolina	N	0	0	N	N	N	0	0	N	N
South Carolina	—	6	16	365	452	—	0	3	24	29
Virginia	N	0	0	N	N	—	0	0	—	—
West Virginia	—	5	83	341	366	—	0	8	21	25
**E.S. Central**	9	16	50	**1,180**	**1,408**	—	1	3	**91**	**121**
Alabama	—	0	0	**106**	**42**	—	0	0	**15**	**10**
Kentucky	—	3	9	179	226	—	0	1	9	23
Mississippi	—	0	0	**175**	**148**	—	0	0	**25**	**14**
Tennessee	9	12	47	720	992	—	1	3	42	74
**W.S. Central**	13	26	188	1,507	2,090	2	2	12	147	192
Arkansas	8	3	14	169	228	—	0	3	13	14
Louisiana	—	4	18	235	259	—	0	4	30	25
Oklahoma	N	0	0	N	N	—	0	0	—	—
Texas	5	20	156	1,103	1,603	2	2	11	104	153
**Mountain**	5	25	53	**1,442**	**1,963**	—	2	6	**125**	**150**
Arizona	2	11	27	**510**	767	—	1	3	**41**	55
Colorado	—	8	19	408	494	—	0	3	36	38
Idaho	N	0	0	N	N	—	0	0	—	—
Montana	—	0	0	**22**	**20**	N	0	0	N	N
Nevada	—	0	0	**78**	**124**	—	0	0	**8**	**6**
New Mexico	3	5	14	251	329	—	0	4	19	24
Utah	—	3	8	144	206	—	0	3	19	27
Wyoming	—	0	3	29	23	—	0	1	2	—
**Pacific**	—	4	10	209	145	—	1	2	28	12
Alaska	—	2	8	139	138	—	0	2	22	10
California	N	0	0	N	N	N	0	0	N	N
Hawaii	—	1	6	70	7	—	0	1	6	2
Oregon	N	0	0	N	N	N	0	0	N	N
Washington	N	0	0	N	N	N	0	0	N	N

**Territories**										
American Samoa	N	0	0	N	N	—	0	0	—	—
C.N.M.I.	—	—	—	—	—	—	—	—	—	—
Guam	—	0	0	—	—	—	0	0	—	—
Puerto Rico	—	0	0	—	—	—	0	0	—	—
U.S. Virgin Islands	—	0	0	—	—	—	0	0	—	—

C.N.M.I.: Commonwealth of Northern Mariana Islands.

U: Unavailable. —: No reported cases. N: Not reportable. NN: Not Nationally Notifiable. Cum: Cumulative year-to-date counts. Med: Median. Max: Maximum.

* Case counts for reporting year 2012 and 2013 are provisional and subject to change. For further information on interpretation of these data, see http://wwwn.cdc.gov/nndss/document/ProvisionalNationaNotifiableDiseasesSurveillanceData20100927.pdf. Data for TB are displayed in Table IV, which appears quarterly.

† Includes drug resistant and susceptible cases of invasive *Streptococcus pneumoniae* disease among children <5 years and among all ages. Case definition: Isolation of *S. pneumoniae* from a normally sterile body site (e.g., blood or cerebrospinal fluid).

